# Can a subgroup at high risk for LRR be identified from T1-2 breast cancer with negative lymph nodes after mastectomy? A meta-analysis

**DOI:** 10.1042/BSR20181853

**Published:** 2019-09-20

**Authors:** Gongling Peng, Zhuohui Zhou, Ming Jiang, Fan Yang

**Affiliations:** Department of Thyroid and Breast Surgery, The Central Hospital of Wuhan, Tongji Medical College, Huazhong University of Science and Technology, 26 Shengli Street, Wuhan 430014, China

**Keywords:** breast cancer, local recurrence, PMRT, risk factors

## Abstract

**Purpose:** To identify a subgroup at high risk for loco-regional recurrence (LRR) from T1-2 breast cancer with negative lymph nodes (N0) after mastectomy by using a meta-analysis.

**Methods and materials:** Published studies on the relationship between clinical features and LRR of breast cancer were identified from public databases, including PubMed, EMBASE, and the Cochrane Library. High-risk features for LRR in this patient population were defined based on the pooled results of meta-analysis.

**Results:** For the meta-analysis, a total of 11244 breast cancers with pT1-2N0 after mastectomy from 20 publications were included for analysis. The pooled results indicated that age (hazard ratio (HR) 1.77, *P*=0.001), lymphovascular invasion (LVI) (HR 2.23, *P*<0.001), histologic grade (HR 1.66, *P*<0.001), HER2 status (HR 1.65, *P*=0.027), menopausal status (HR 1.36, *P*=0.015), and surgical margins (HR 2.56, *P*=0.014) were associated with a significantly increased risk of developing LRR in this patient population group, but not for tumor size (HR 1.32, *P*=0.23), systematic therapy (HR 1.67, *P*=0.20), and hormonal receptor status (HR 1.04, *P*=0.73).

**Conclusion:** In the current study, patients with young age, positive LVI, high histologic grade, HER-2 positive, premenopausal, and positive surgical margins have an increased risk of developing LRR. Further prospective trials are needed to clearly define the role of adjuvant postmastectomy radiotherapy in T1-2N0 breast cancer at high risk of developing LRR.

## Introduction

Post-mastectomy radiation therapy (PMRT) has generally not been recommended as a routine part of treatment for T1–T2 breast cancer with negative lymph node (N0) after mastectomy, due to the low loco-regional recurrence (LRR) rates in this patient group as a whole [1,2]. A recent meta-analysis conducted by the Early Breast Cancer Trialists Collaborative Group (EBCTCG) [3] also demonstrated that PMRT did not significantly reduce the 10-year LRR first [3.0% (no RT) versus 1.6% (RT)] in node negative breast patients receiving mastectomy. However, it is becoming increasingly clear that breast cancer represents a heterogeneous group of diseases. And multiple retrospective studies have identified a number of potential risk factors, such as age, tumor size, lymphovascular invasion (LVI), histologic grade, and margin status, for LRR after mastectomy, and patients with certain risk factors might have LRR risks in excess of 20% [4,5]. As a result, patients with multiple risk factors of LRR could derive significant benefit from PMRT in terms of LRR, and a potential survival benefit.

Currently, no consensus has been archived regarding what constitutes ‘high risk’ in the absence of lymph node metastases in this patient group [6,7]. For example, tumor size, histologic grade, and LVI were statistically significant high risk for LRR in T1-2N0 breast cancer after mastectomy in the Truong et al.’s study [8,9], while only tumor size, but not for histologic grade and LVI, was regarded as high risk feature for LRR in Mamtani et al.’s study [10]. As a result, we perform the present meta-analysis to pool the controversial results from multiple included studies, which could increase the statistical power to detect an effect and resolve uncertainty when reports disagree, and aim to identify risk factors for LRR in T1-2N0 breast cancer after mastectomy by using a meta-analysis.

## Materials and methods

We performed the meta-analysis according to the Preferred Reporting Items for Systematic Reviews and Meta-Analyses (PRISMA) statement guidelines 2009 [11].

### Search strategy and study selection

We conducted a comprehensive literature search of public databases including PubMed, EMBASE, and the Cochrane Library (up to 31 March 2018). Relevant search keywords included the following: ‘breast cancer’, ‘mastectomy’, ‘loco-regional disease recurrence’, and ‘lymph node negative’. No language restriction was administered. We also conducted a manual search of conference proceedings. All results were input into Endnote X8 reference software (Thomson Reuters, Stamford, CT, U.S.A.) for duplication exclusion and further reference management.

Clinical trials that met the following criteria were included: (1) prospective or retrospective studies involving early stage (T1-2) breast cancer patients with negative lymph node after mastectomy; (2) available data regarding the relationship between clinical factors and LRR of breast cancer after mastectomy; if multiple publications of the same trial were retrieved or if there was a case mix between publications, only the most recent publication (and the most informative) was included.

### Data extraction and statistical analysis

Two independent investigators conducted the data abstraction, and any discrepancy between the reviewers was resolved by consensus. The following information was extracted for each study: first author’s name, year of publication, number of enrolled subjects, surgical types, median follow-up, LRR definitions, and LRR rate.

A formal meta-analysis was conducted using Comprehensive Meta Analysis software (Version 2.0). The outcome data were pooled and reported as hazard ratio (HR). The primary outcome of interest was the relationship between clinical factors and LRR of breast cancer after mastectomy.

All statistical analyses were performed by using Version 2 of the Comprehensive MetaAnalysis program (Biostat, Englewood, NJ). Between-study heterogeneity was estimated using the χ^2^-based Q statistic [12]. The *I*^*2*^ statistic was also calculated to evaluate the extent of variability attributable to statistical heterogeneity between trials. A statistical test with a *P*-value less than 0.05 was considered significant. To assess the stability of results, sensitivity analysis was carried out by sequential omission of individual studies.

## Results

**Figure 1:**
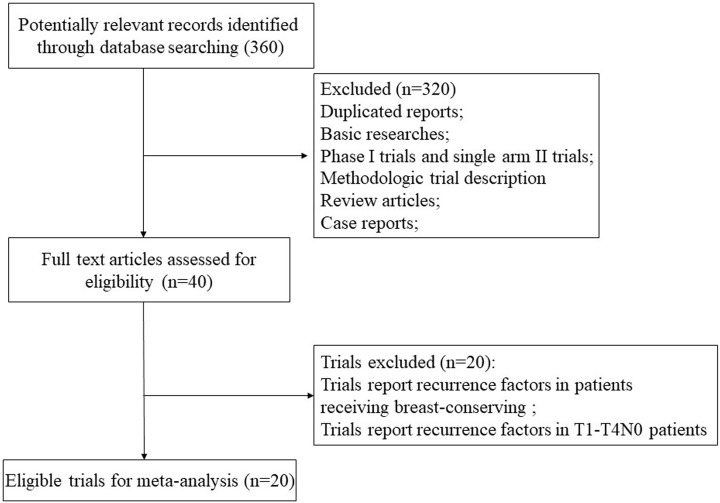
PRISMA flow diagram

We initially found 360 relevant citations in early stage breast cancer. After excluding review articles, Phase I studies, case reports, editorial, letters, commentaries, meta-analyses, and systematic review (Figure 1), a total of 20 retrospective studies were finally included for analysis in the present studies. In all, 11244 breast cancer with pT1-2N0 breast cancers after mastectomy from 20 published studies were included for analysis [4,8–10,13–28]. Table 1 listed the baseline characteristics of the patients and studies. The incidence of 10-year local recurrence after mastectomy in pT1-2N0M0 breast cancer ranged from 2.1 to 12.8%, and the high-risk factors of pT1-2N0 breast cancer are listed in Table 2.

**Table 1:** Baseline characteristics of 20 included studies

Author/year	Series type	Center	Years	T stage	*n*	Age	Surgical type	Chemotherapy, %	Hormone therapy, %	LRR definition	Median follow-up, years	LRR, %
**Ahlborn/1988**	R	Columbia University College	1975–1985	T1-2	346	28–90	MRM	0	0	Recurrence on CW	3.9	4-y, 4%
**Janni et al./2000**	R	Ludwig-Maximilians- Universtitaet	1963–1998	T1-2	114	<75	MRM and AC+PMRT	0	NR	Isolated LRR, LRR with or without DF	6	10-y, 14%
					804	<75	MRM and AC	0	NR			10-y, 4%
**Voogd/2001**	R	EORTC and DBCG	1980–1989	T1-2	535	<70	MRM and AC I/II	NR	NR	Isolated LRR, LRR with or without DF	9.8	10-y, 9%
**Wallgren et al./2003**	R	IBCSG trials	1981–1985	T1-3 (T3 2.1%)	1275	NR	MRM	66	NR	Isolated LRR, LRR with or without DF, DF	14.5	10-y, 12.8%
**Colleoni et al./2005**	R	IBCSG trials	1978–1999	T1-3	2588	Median: 54	M and AC	67	0	Isolated LRR, LRR with or without DF	11	10-y, 10%
**Truong et al./2005**	R	BCCA	1989–1999	T1-2	1505	24–95	M	14.1	29.9	LRR with or without DF	7	10-y, 7.8%
**Jagsi et al./2005**	R	MGH	1980–2000	T1-3 (T3 2.85%	877	Any	MRM and AC	8.4	16.9	Isolated LRR, LRR with or without DF	8.3	10-y, 6%
**Truong et al./2005**	R	BCCA	1989–1999	T1-2	763	24–89	M	27.8	59	LRR with or without DF	7	NR
**Buchanan/2006**	P	MSCC	1995–1999	T1	325	Any	M and AC I/II	NR	NR	Isolated LRR, LRR with or without DF	6	5-y, 4%
**Yildrim et al./2007**	R	Ankara Oncology Training and Research Hospital	1990–2004	T1-2	502	<70	MRM and AC I/II/III	56	43	Isolated LRR	6.4	10-y, 3%
**Karlsson et al./2007**	R	IBCSG trials	1978–1999	T1-3	2588	NR	M and AC, adjuvant systematic therapy	NR	NR	Isolated LRR, LRR with or without DF	14	10-y, 10%
**Mamouna et al./2010**	R	B-14/B-20	1982–1993	T1-3 (T3 5%)	505	NR	M	0	100	Isolated LRR	12.5	10-y, 6.1%
**Sharma et al./2010**	R	M.D. Anderson Cancer Center	1997–2002	T1-2	753	NR	M	NR	NR	Isolated LRR, LRR with or without DF	7.47	10-y, 2.1%
**Abi-Raad et al./2011**	R	MGH	1980–2004	T1-2	1136	Any	MRM and AC	6.8	23.8	Isolated LRR, LRR with or without DF	9	10-y, 5.2%
**Selz et al./2012**	R	Hoôpital René Huguenin	2001–2008	T1-3 (3.5% T3)	191	Median: 56	MRM and AC I/II+PMRT	68.1	73.3	LRRFS	4.7	5-y, 2.1%
					508	Median: 63	MRM and SLNB	20.5	62.6			5-y, 2.6%
**Truong et al./2014**	R	BCCA and MGH	1998–2009	T1-2	1994	22–97	M	11.5	48.2	Isolated LRRFS (excluded if DF within 4 months of LRR)	4.3	5-y, 1.75%
**Jwa et al./2015**	R	Soonchunhyang University College of Medicine	2002–2011	T1-2	390	37–87	MRM and AC	47.60%	NR	Isolated LRR, LRR with or without DF	5.6	5-y, 2.6%
**Li et al./2017**	R	Fujian Medical University Cancer Hospital	2001–2008	T1-2	353	NR	MRM and AC	NR	NR	Isolated LRR, LRR with or without DF	9.6	5-y, 11%
**Mamtani et al./2017**	R	MSKCC	2006–2011	T1-2	657	33–86	M	14%	32%	Isolated LRR, LRR with or without DF	5.6	5-y, 4.7%
**Frandsen et al./2017**	R	Huntsman Cancer Hospital	1978–2014	T1-2	38	25–40	MRM and AC+PMRT	55.30%	2.60%	Isolated LRR, LRR with or without DF	6	10-y 0%
					181	18–40	MRM and AC	35.90%	12.70%	Isolated LRR, LRR with or without DF	4.6	10-y 10%

Abbreviations: AC, axillary clearance (followed by levels cleared); BCCA, British Columbla Cancer Agency; DF, distant failure; IBCSG, International Breast Cancer Study Group; LRRFS, LRR-free survival; M, mastectomy; MGH, Massachusetts General Hospital; MRM, modified radical mastectomy; MSKCC, Memorial Sloan Cancer Center; NR, not reported; P, prospective; R, retrospective.

**Table 2:** LRR after mastectomy in T1-2 N0 breast cancer

Author/year	Overall: LRR	High-risk: 10-year LRR	Low risk: 10-year	High-risk definition	Low-risk definition
**Ahlborn/1998**	4-year, 4%	NR	NR	NR	NR
**Janni et al./2000**	5-year, 8.8%	NR	NR	NR	NR
**Voogd/2001**	10-year, 9%	15%	8%	LVI	no LVI
**Wallgren et al./2003 (premenopausal)**	10-year, 12.8%	16%	8%	LVI, T ≥ 2 cm	no LVI, T < 2 cm
**Wallgren et al./2003 (postmenopausal)**		19%	8%	LVI	no LVI
**Colleoni et al./2005**	10-year, 10%	NR	NR	NR	NR
**Truong et al./2005**	10-year, 7.8%	21.20%	4.50%	LVI, grade 3	grade 1–2, age ≥50
**Truong et al./2005**	NR	7-year, 19.5%	7 year, 3.4%	LVI, age <50	No LVI, age ≥50
**Jagsi et al./2005**	10-year, 6%	10.0%, 17.9%, 40.6% for 1, 2 and 3 risk factors	1.20%	close margins, T > 2 cm, premenopausal, and LVI	no risk factor
**Buchanan/2006**	5-year, 4%	NR	NR	age ≤ 35, LVI, and multi-centricity	No risk
**Karlsson et al./2007 (premenopausal)**	14-year, 12.5%	14.70%	10.90%	1–10 uninvolved nodes	≥19 uninvolved nodes
**Karlsson et al 2007 (postmenopausal)**	14-year, 8.2%	11.60%	6.20%	1–10 uninvolved nodes	≥19 uninvolved nodes
**Yildrim et al./2007 (≤40 years)**	10-year, 3%	NR	NR	T >2 cm and LVI	0–1 risk factor
**Yildrim et al./2007 (>40)**		NR	NR	T >3 cm, high grade, and LVI	0–2 risk factors
**Mamouna et al./2010**	10-year, 6.1%	16.80%	2.30%	high 21-gene recurrence score	Low 21 gene recurrence score
**Sharma et al./2010**	10-year, 2.1%	18.60%	1.00%	T2, ≤40 years	T1-2, >40 years
**Abi-Raad et al./2011**	10-year, 5.2%	19.70%	3.30%	LVI, positive margins, T ≥ 2 cm, age ≤50 years	No LVI, age >50, negative margins, size <2 cm, systemic therapy
**Selz et al./2012**	5-year 2.6%	5-year, 15.1%^1^	5-year, 2.6%^1^	Ki67 > 20%	Ki67 ≤ 20%
**Truong et al./2014**	5-year 1.75%	5-year, 12.5%	5-year, 1.1%	TNBC, close or positive margins	negative margin, luminal;
**Jwa et al./2015**	5-year 2.6%	5-year, 14%^1^	5-year, 0%, 5% for 0 and 1 risk^1^ factors	age ≤ 50, systematic chemotherapy (2 risk factors)	0–1 risk factors
**Li et al./2017**	5-year 11%	5-year, 24.3%	5-year, 8.4%	Age < 40 years, T ≥ 4.5 cm, number of resected nodes ≤ 10	0–1 risk factor
**Mamtani et al./2017**	5-year 4.7%, 5.3%	5-year, 9.4% for ≥4 risk factors	3.80%	age < 40 years, multifocal or multicentric tumor, LVI, central or medial tumor location, and high nuclear grade (≥2 risk factors)	0–2 risk factor
**Frandsen et al./2017**	10-year 10%	28.00%	6.70%	LVI	No LVI

Abbreviations: AC, axillary clearance (followed by levels cleared); BCCA, British Columbla Cancer Agency; DF, distant failure; IBCSG, International Breast Cancer Study Group; LRRFS, LRR-free survival; MGH, Massachusetts General Hospital; M, mastectomy; MRM, modified radical mastectomy; MSKCC, Memorial Sloan Cancer Center; NR, not reported; P, prospective; R, retrospective.

1 Actuarial failure rate or calculated from local disease-free survival obtained by Kaplan–Meier method rather than cumulative incidence of LRR.

### Age

**Figure 2:**
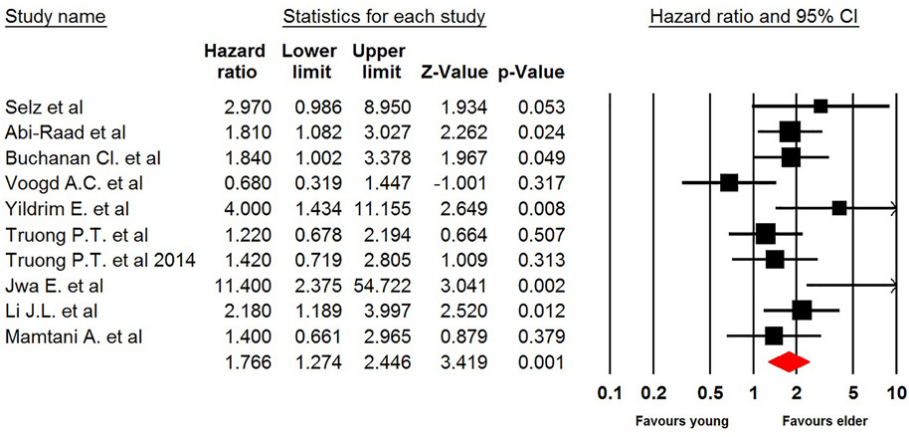
Meta-analysis of LRR rate in young versus elder patients

Ten included trials reported the association between age and LRR among pT1-2N0 breast cancer after mastectomy. The pooled results showed that young breast cancer patients had a significantly increased risk of developing LRR in comparison with elder patients (HR 1.77, 95% CI: 1.27–2.45, *P*=0.001, Figure 2). We also did sensitivity analysis to examine the stability and reliability of pooled HRs by sequential omission of individual studies. The results indicated that the significance estimate of pooled HRs was not significantly influenced by omitting any single study. Similar results were observed in subgroup analysis according to median follow-up time (long follow-up: HR 1.45, 95% CI: 1.04–2.00, *P*=0.027; short follow-up: HR 1.69, 95% CI: 1.43–2.64, *P*<0.001, Supplementary Figure S1).

### LVI

**Figure 3:**
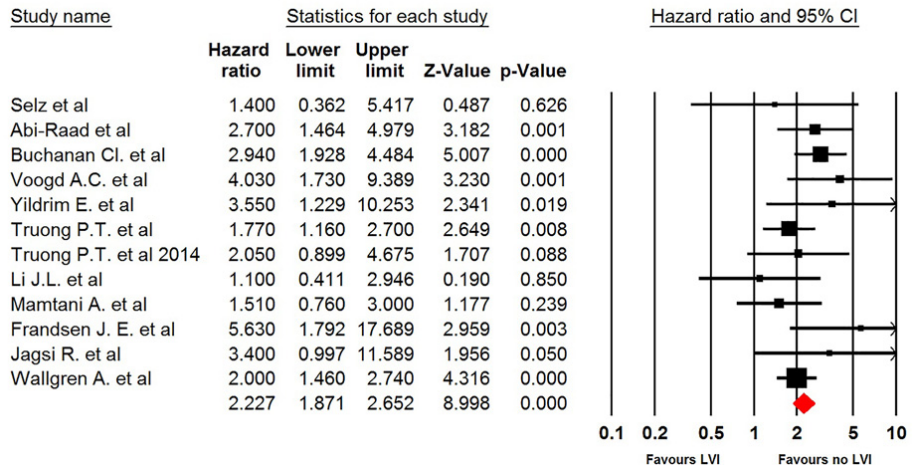
Meta-analysis of LRR rate in LVI versus no LVI

Twelve studies contributed to the pooled analysis. Breast cancer patients with positive LVI were associated with a significantly increased risk of developing LRR compared with negative LVI (HR 2.23, 95% CI: 1.87–2.65, *P*<0.001, Figure 3). No significant heterogeneity across the studies was detected (*I^2^* = 0; *P*=0.37). Sensitivity analysis indicated that the significance estimate of pooled HRs was not significantly influenced by omitting any single study. Similar results were observed in subgroup analysis according to median follow-up time (long follow-up: HR 2.21, 95% CI: 1.76–2.77, *P*<0.001; short follow-up: HR 2.25, 95% CI: 1.71–2.96, *P*<0.001, Supplementary Figure S2).

### Histologic grade

**Figure 4:**
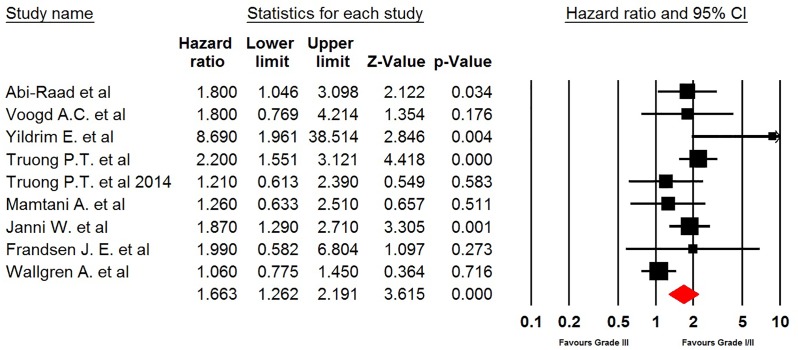
Meta-analysis of LRR rate in Grade III versus Grade I/II

Nine studies contributed to the pooled analysis. Breast cancer patients with Grade 3 histologic type were associated with a significantly increased risk of developing LRR when compared with Grades 1–2 histologic type (HR 1.66, 95% CI: 1.26–2.19, *P*<0.001, Figure 4). No heterogeneity across the studies was detected. Sensitivity analysis indicated that the significance estimate of pooled HRs was not significantly influenced by omitting any single study. Similar results were observed in subgroup analysis according to median follow-up time (long follow-up: HR 1.63, 95% CI: 1.35–1.98, *P*<0.001; short follow-up: HR 1.45, 95% CI: 1.02–2.09, *P*=0.041, Supplementary Figure S3).

### HER-2 status

**Figure 5:**
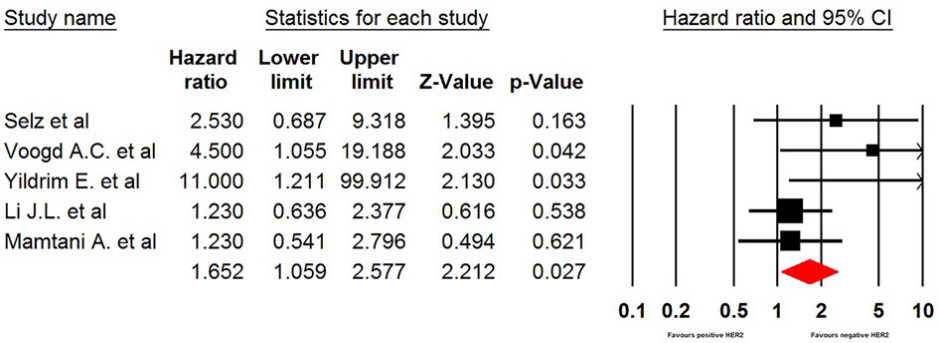
Meta-analysis of LRR rate in HER2 positive versus HER2 negative

In five studies, data about HER-2 status and LRR risk were available. Breast cancer with positive HER-2 status was associated with an increased risk of developing LRR in comparison with negative HER-2 status (HR 1.65, 95% CI: 1.06–2.58, *P*=0.027, Figure 5). Significant heterogeneity was observed. Sensitivity analysis indicated that the significance estimate of pooled HRs was significantly influenced by omitting any single study.

### Menopausal status

Five studies contributed to the pooled analysis. Our results showed that menopausal status was significantly associated with LRR in T1-2N0 breast cancer patients. Premenopausal breast cancer was associated with an increased risk of developing LRR in comparison with postmenopausal breast cancer patients (HR 1.36, 95% CI: 1.06–1.74, *P*=0.015, Supplementary Figure S4). Significant heterogeneity was observed. Sensitivity analysis indicated that the significance estimate of pooled HRs was significantly influenced by omitting any single study.

### Surgical margins

In three studies with surgical data available, breast cancer with positive/close surgical margins was associated with an increased risk of developing LRR in comparison with negative surgical margins (HR 2.56, 95% CI: 1.21–5.41, *P*=0.014, Supplementary Figure S5). Significant heterogeneity was observed. Sensitivity analysis indicated that the significance estimate of pooled HRs was significantly influenced by omitting any single study.

### Systematic therapy

Eight studies contributed to the pooled analysis. There was no significant risk difference of LRR in breast cancer receiving systematic therapy (chemotherapy/hormonal therapy) or not (HR 1.67, 95% CI: 0.77–3.50, *P*=0.20, Supplementary Figure S6). Significant heterogeneity was observed.

### Tumor size

Thirteen studies contributed to the pooled analysis. Tumor size was not a significant risk factor for LRR in T1-2 breast cancer with negative lymph node after mastectomy (HR 1.32, 95% CI: 0.85–2.05, *P*=0.23, Supplementary Figure S7). No significant heterogeneity was observed.

### Hormonal receptor status

Thirteen studies contributed to the pooled analysis. Hormonal receptor status was not a significant risk factor for LRR in T1-2 breast cancer with negative lymph node after mastectomy (HR 1.04, 95% CI: 0.76–1.44, *p*=0.73, Supplementary Figure S8). No significant heterogeneity was observed.

## Discussion

Of note, the management of early-stage women with breast cancer after mastectomy is a heterogeneous disease because different subgroups demonstrate significant variation in the risk for recurrence and survival, and in selected women with high risks; the 5-year LRR can be as high as 20% [29,30]. It has been reported that effective local control is associated with improved overall survival, especially for early-stage breast cancer patients [31]. As a result, accurately predicting the women who are at highest risk for recurrence after mastectomy will identify those who might benefit from more aggressive locally adjuvant treatment. During the past decades, although multiples studies have been conducted to investigate the risk factors associated with local recurrence in T1-2 N0 breast cancer after mastectomy, but the results are controversial. In 2005, the ninth St Gallen expert panel agreed that radiation therapy could reduce replacement in the early breast cancer after breast conserving surgery, and the balance between beneficial and harmful effects of PMRT depends on the risk of local recurrence [32], the age of the patient, the efficacy of systemic therapies (especially endocrine agents) and competing causes of morbidity and mortality. However, no quantitative evaluations of clinic-pathological risk features of LRR have been previously investigated. As a result, we conduct the present meta-analysis to comprehensively evaluate the risk factors for local recurrence in early-stage breast cancer after mastectomy.

To the best of our knowledge, our meta-analysis is the largest and most comprehensive systematic review to specially investigate risk factors for local recurrence in pT1-2 N0 breast cancer after mastectomy. A total of 11244 pT1-2 breast cancers with negative lymph nodes after mastectomy from 15 publications were included for analysis. The pooled results have demonstrated that T1-2 breast cancer with positive LVI (HR 2.23, *P*<0.001) or surgical margins (HR 2.56, *P*=0.014) has increased twice the risk of developing LRR. Consistent with our findings, previous research even found that the LRR of patients with negative lymph node disease and LVI who do not receive PMRT is worse than that of patients with node-positive disease receiving adjuvant radiotherapy [33]. Additionally, the ninth St Gallen expert panel also accepted LVI as a discriminatory feature of increased risk for T1-2 breast cancer with negative lymph node. As for the risk of patients with a close or positive surgical margin after mastectomy, most of published data identified a close or positive surgical margin would increase the risk of chest wall failure [34,35]. In clinical practice today, patients with a close or positive surgical margin after mastectomy are likely to be treated with chest wall irradiation. Our study also suggests that age (HR 1.77, *P*=0.001), histologic grade (HR 1.66, *P*<0.001), HER2 status (HR 1.65, *P*=0.027), menopausal status (HR 1.36, *P*=0.015) are risk predictors for LRR in this patient population group.

Several previous research have reported different results in terms of systemic chemotherapy as a risk factor for LRR. Truong et al. [8] found that no systemic therapy was associated with increased risk of LRR compared with systemic therapy (14.1% chemotherapy alone, 29.9% hormone therapy alone, 6.7% both) in patients with pT1-2N0 cancer (HR 1.87; *P*=0.01), while Wallgren et al. [25] analyzed 1275 patients with node-negative disease and found that the LRR risk increased significantly without adjuvant chemotherapy. In the present study, the pooled results showed that systematic therapy (HR 1.67, *P*=0.20) is not a significant risk factor for LRR. The implications of tumor size and hormonal receptor status on recurrence remain controversial. For example, Abi-Raad et al. [15] reported tumor size is associated with increased LRR risk, while it is not a significant risk factor in Jwa et al. study [27]. In the present study, both tumor size (HR 1.32, *P*=0.23) and hormonal receptor status (HR 1.04, *P*=0.73) are not significantly associated with LRR in breast cancer after mastectomy. Consistent with our pooled results, the ninth St Gallen expert panel also regarding age, histologic grade, LVI, and HER2 status as risk factors to identify node negative early breast cancer patients at high risk for LRR [32]. Based on these findings, for early-stage breast cancer with high risk for local recurrence, such as young age, high histologic grade, positive LVI positive, premenopausal and/or positive surgical margins, additional adjuvant local therapy might be warranted for this patient population group in order to reduce the LRR risk.

There are several limitations that need to be mentioned. First, this is a meta-analysis at study level, thus individual patient information is not available from the publication. Second, the application of formal meta-analytic methods to observational studies has been controversial. One of the most important reasons for this is that the designs and populations of the studies are diverse, and that these differences may influence the pooled estimates. However, when no head-to-head comparison data are available for combination therapy versus mono-therapy, a meta-analysis of observational studies is one of the few methods for assessing risk factors for local recurrence. Third, all included studies are retrospective clinical studies, treatment regimen and follow-up time of included patients are significantly different, thus all of these would increase the heterogeneity among included studies.

## Conclusion

Despite these limitations, the result of this meta-analysis for the first time clearly demonstrates that early breast patients after mastectomy with young age, positive LVI, high histologic grade, HER-2 positive, premenopausal, and/or surgical margins positive have an increased risk of developing LRR, and additional local radiotherapy might be warranted for this subset group population. Further prospective trials are needed to clearly define the role of adjuvant postmastectomy radiotherapy in pT1-2N0 breast cancer with high risk for developing LRR.

## Supporting information

**Supplementary Figure S1:** Sub-group meta-analysis of loco-regional recurrence rate in young versus elder patients

**Supplementary Figure S2:** sub-group meta-analysis of loco-regional recurrence rate in LVI versus no LVI

**Supplementary Figure S3:** sub-group meta-analysis of loco-regional recurrence rate in Grade III versus Grade I/II

**Supplementary Figure S4:** Meta-analysis of loco-regional recurrence rate in pre-menopausal versus post-menopausal patients

**Supplementary Figure S5:** Meta-analysis of loco-regional recurrence rate in close/positive margins versus negative margins

**Supplementary Figure S6:** Meta-analysis of loco-regional recurrence rate in patients received with or without systematic therapy

**Supplementary Figure S7:** Meta-analysis of loco-regional recurrence rate in T1 versus T2 patients

**Supplementary Figure S8:** Meta-analysis of loco-regional recurrence rate in HR positive versus HR negative patients

## Abbreviations

HR: hazard ratio
LRR: loco-regional recurrence
LVI: lymphovascular invasion
PMRT: post-mastectomy radiation therapy
RT: radiation therapy
95% CI: 95% confidence interval
