# Supplementary material for: Can a subgroup at high risk for LRR be identified from T1-2 breast cancer with negative lymph nodes after mastectomy? A meta-analysis
Source: Biosci Rep. 2019 Sep 20;39(9):BSR20181853. doi: 10.1042/BSR20181853 (PMC6753322; doi:10.1042/BSR20181853)

Supplementary Figure Legends

Supplemental figure 1: Sub-group meta-analysis of loco-regional recurrence rate in young versus elder patients

Supplemental Figure 2 sub-group meta-analysis of loco-regional recurrence rate in LVI versus no LVI

Supplemental figure 3: sub-group meta-analysis of loco-regional recurrence rate in Grade III versus Grade I/II

Supplemental figure 4: Meta-analysis of loco-regional recurrence rate in pre-menopausal versus post-menopausal patients

Supplemental figure 5: Meta-analysis of loco-regional recurrence rate in close/positive margins versus negative margins

Supplemental figure 6: Meta-analysis of loco-regional recurrence rate in patients received with or without systematic therapy

Supplemental figure 7: Meta-analysis of loco-regional recurrence rate in T1 versus T2 patients

Supplemental figure 8: Meta-analysis of loco-regional recurrence rate in HR positive versus HR negative patients

Group by follow-up	Study name	Hazard ratio	Lower limit	Upper limit	Z-Value	p-Value
long	Abi-Raad et al	1.810	1.082	3.027	2.262	0.024
long	Voogd A.C. et al	0.680	0.319	1.447	-1.001	0.317
long	Yildrim E. et al	4.000	1.434	11.155	2.649	0.008
long	Truong P.T. et al	1.220	0.678	2.194	0.664	0.507
long		1.446	1.044	2.004	2.216	0.027
short	Selz et al	2.970	0.986	8.950	1.934	0.053
short	Buchanan Cl. et al	1.840	1.002	3.378	1.967	0.049
short	Truong P.T. et al 2014	1.420	0.719	2.805	1.009	0.313
short	Jwa E. et al	11.400	2.375	54.722	3.041	0.002
short	Li J.L. et al	2.180	1.189	3.997	2.520	0.012
short	Mamtani A. et al	1.400	0.661	2.965	0.879	0.379
short		1.938	1.426	2.636	4.222	0.000
Overall		1.689	1.351	2.113	4.593	0.000

Statistics for each study

Hazard ratio and 95% CI

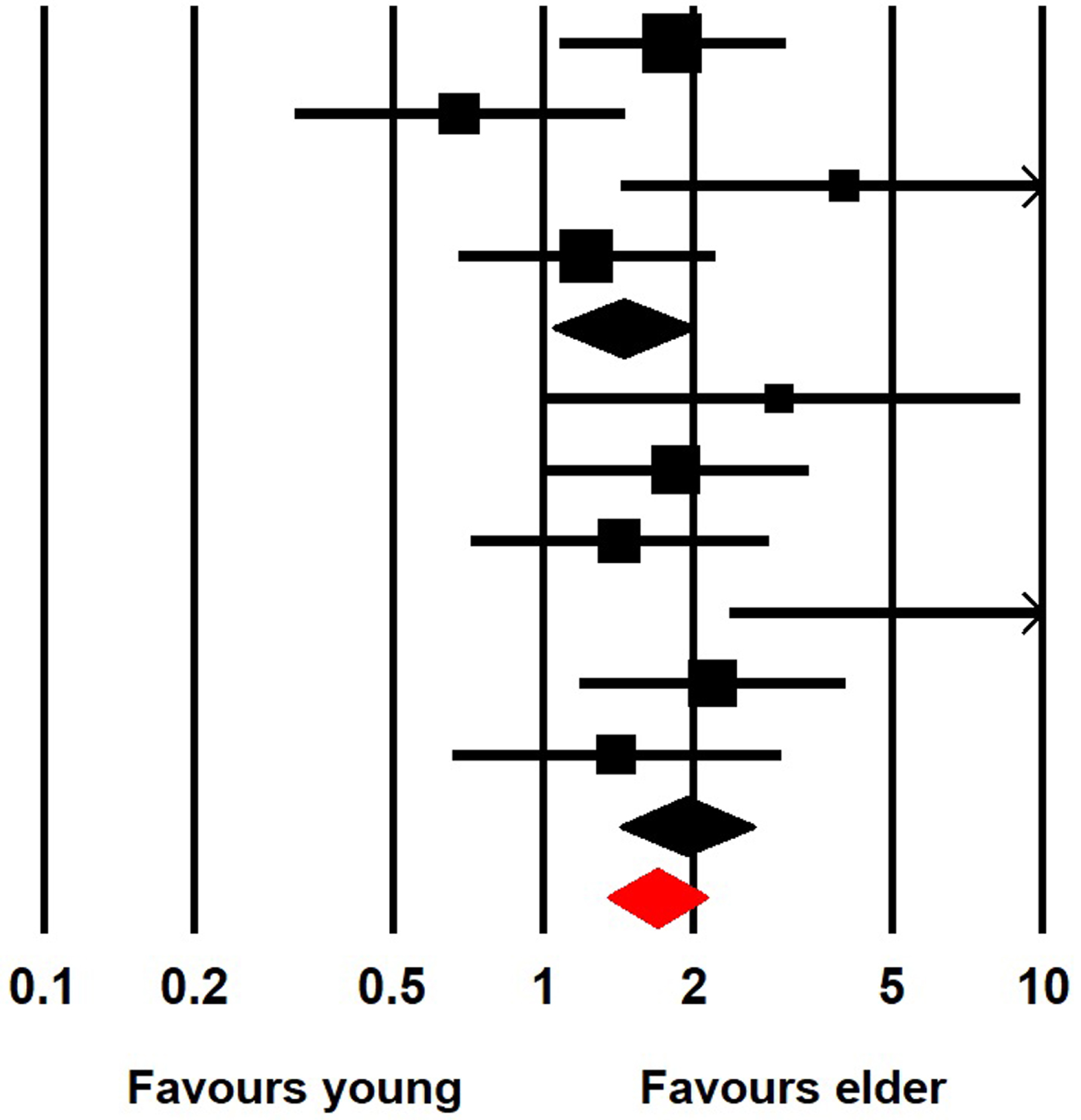

Group by follow-up	Study name	Hazard ratio	Lower limit	Upper limit	Z-Value	p-Value
long	Voogd A.C. et al	4.030	1.730	9.389	3.230	0.001
long	Yildrim E. et al	3.550	1.229	10.253	2.341	0.019
long	Truong P.T. et al	1.770	1.160	2.700	2.649	0.008
long	Frandsen J. E. et al	5.630	1.792	17.689	2.959	0.003
long	Jagsi R. et al	3.400	0.997	11.589	1.956	0.050
long	Wallgren A. et al	2.000	1.460	2.740	4.316	0.000
long		2.211	1.762	2.774	6.852	0.000
short	Selz et al	1.400	0.362	5.417	0.487	0.626
short	Abi-Raad et al	2.700	1.464	4.979	3.182	0.001
short	Buchanan Cl. et al	2.940	1.928	4.484	5.007	0.000
short	Truong P.T. et al 2014	2.050	0.899	4.675	1.707	0.088
short	Li J.L. et al	1.100	0.411	2.946	0.190	0.850
short	Mamtani A. et al	1.510	0.760	3.000	1.177	0.239
short		2.251	1.714	2.957	5.834	0.000
Overall		2.227	1.871	2.652	8.998	0.000

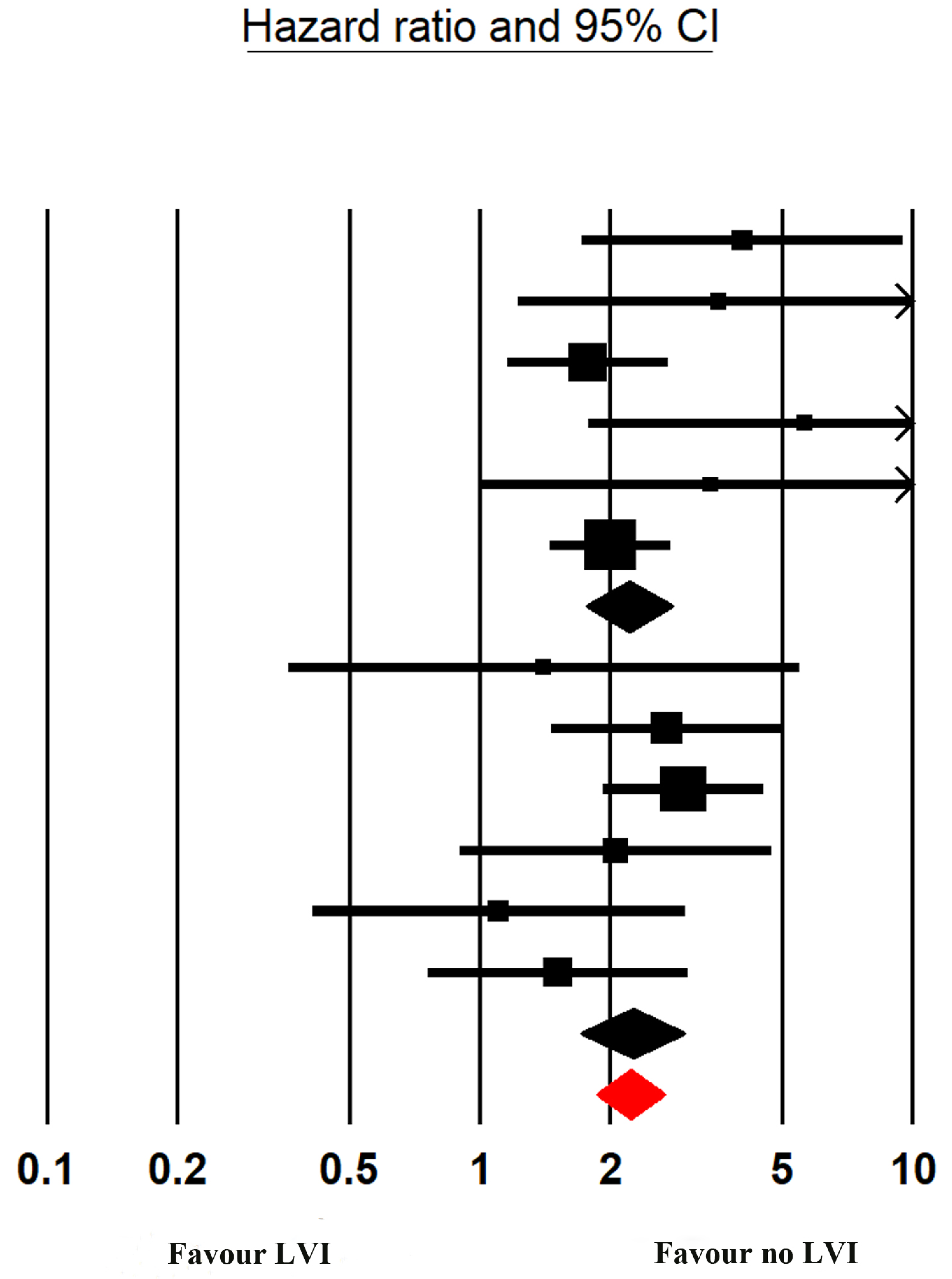

Group by follow-up	Study name	Hazard ratio	Lower limit	Upper limit	Z-Value	p-Value
long	Voogd A.C. et al	1.800	0.769	4.214	1.354	0.176
long	Yildrim E. et al	8.690	1.961	38.514	2.846	0.004
long	Truong P.T. et al	2.200	1.551	3.121	4.418	0.000
long	Janni W. et al	1.870	1.290	2.710	3.305	0.001
long	Frandsen J. E. et al	1.990	0.582	6.804	1.097	0.273
long	Wallgren A. et al	1.060	0.775	1.450	0.364	0.716
long		1.635	1.354	1.975	5.111	0.000
short	Abi-Raad et al	1.800	1.046	3.098	2.122	0.034
short	Truong P.T. et al 2014	1.210	0.613	2.390	0.549	0.583
short	Mamtani A. et al	1.260	0.633	2.510	0.657	0.511
short		1.459	1.016	2.094	2.049	0.041
Overall		1.596	1.350	1.886	5.479	0.000

(The "Statistics for each study" heading spans the Hazard ratio through p-Value columns.)

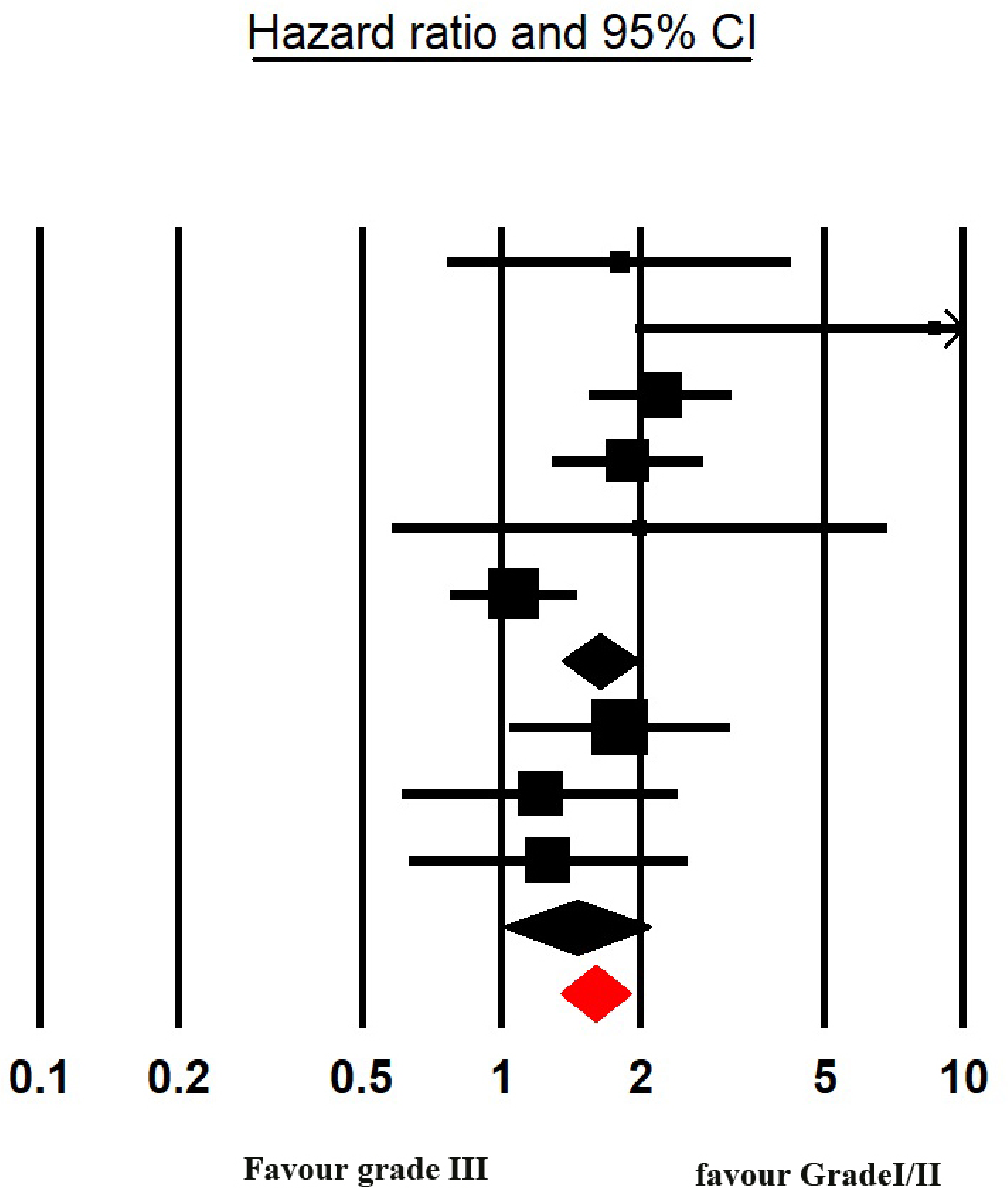

Study name	Statistics for each study					Hazard ratio and 95% CI
	Hazard ratio	Lower limit	Upper limit	Z-Value	p-Value	
Truong P.T. et al 2014	1.820	0.649	5.103	1.138	0.255	
Frandsen J. E. et al	1.300	0.348	4.854	0.390	0.696	
Jagsi R. et al	4.260	2.418	7.505	5.017	0.000	
	2.555	1.207	5.410	2.451	0.014	

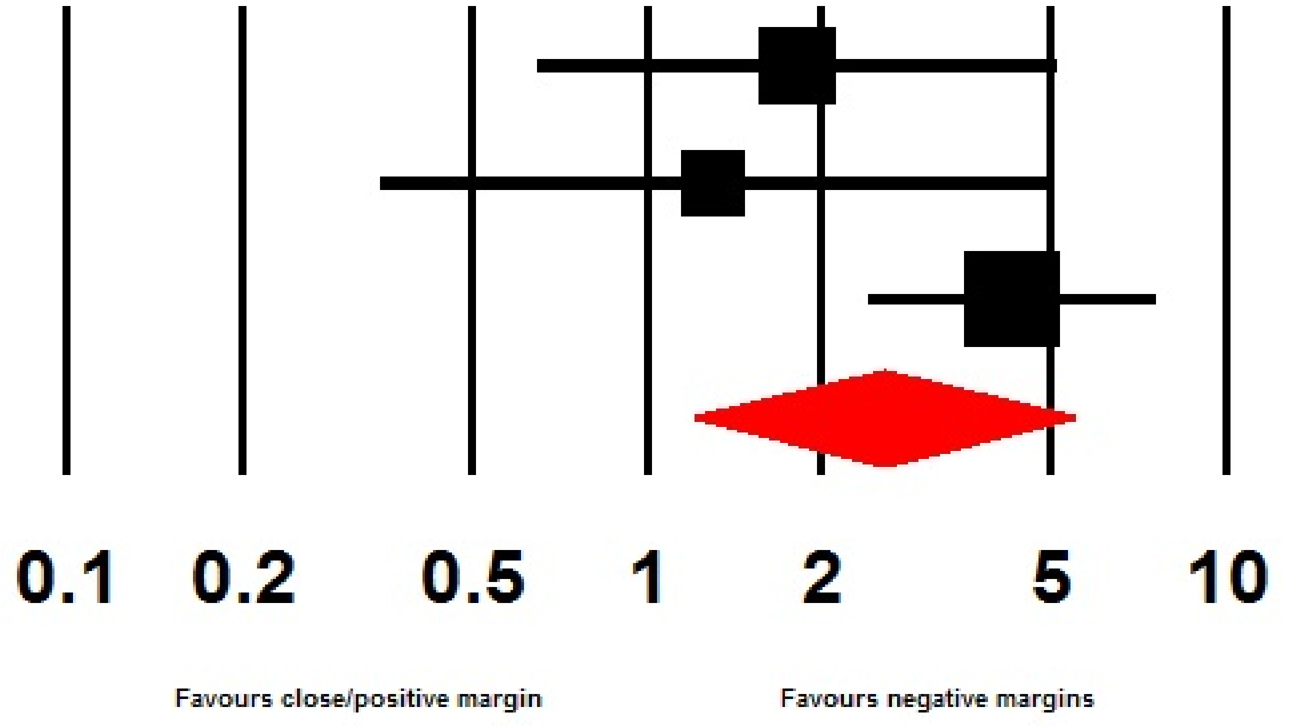

Study name	Hazard ratio	Lower limit	Upper limit	Z-Value	p-Value
Selz et al	2.450	0.801	7.492	1.571	0.116
Abi-Raad et al	0.720	0.419	1.237	-1.189	0.234
Yildrim E. et al	12.800	4.930	33.233	5.237	0.000
Truong P.T. et al	0.420	0.161	1.096	-1.772	0.076
Truong P.T. et al 2014	1.330	0.632	2.799	0.751	0.453
Jwa E. et al	10.200	1.188	87.595	2.117	0.034
Li J.L. et al	0.750	0.364	1.546	-0.779	0.436
Frandsen J. E. et al	1.570	0.471	5.232	0.734	0.463
	1.637	0.766	3.499	1.271	0.204

Statistics for each study

Hazard ratio and 95% CI

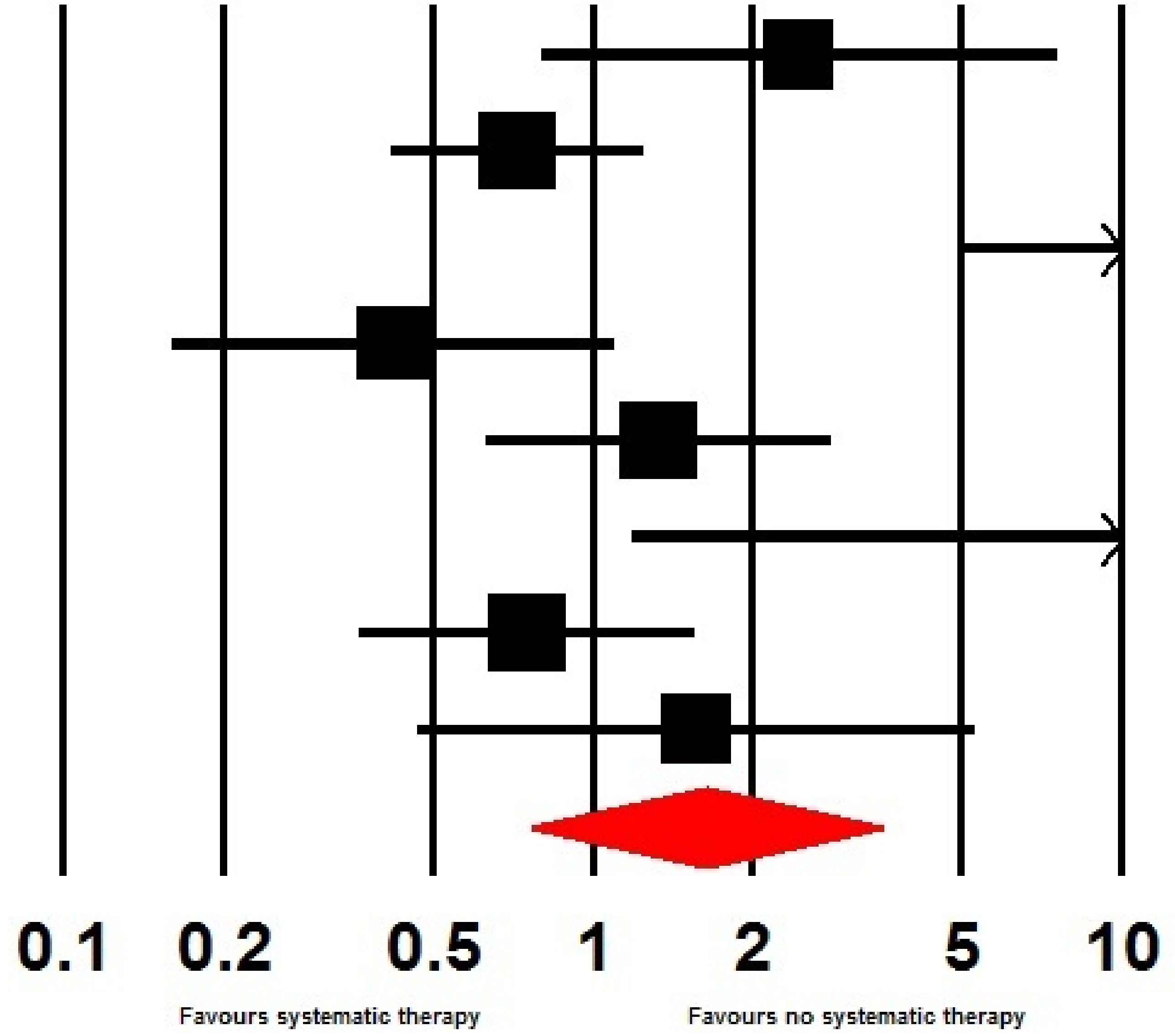

Study name	Hazard ratio	Lower limit	Upper limit	Z-Value	p-Value
Selz et al	1.000	0.612	1.633	0.000	1.000
Abi-Raad et al	1.160	0.661	2.034	0.518	0.605
Buchanan Cl. et al	0.500	0.258	0.970	-2.052	0.040
Voogd A.C. et al	1.620	1.054	2.490	2.200	0.028
Yildrim E. et al	8.340	2.541	27.377	3.497	0.000
Truong P.T. et al	0.680	0.363	1.272	-1.207	0.228
Truong P.T. et al 2014	0.260	0.138	0.491	-4.148	0.000
Li J.L. et al	0.750	0.408	1.378	-0.927	0.354
Mamtani A. et al	2.690	1.341	5.395	2.787	0.005
Janni W. et al	2.340	1.642	3.334	4.705	0.000
Frandsen J. E. et al	1.930	0.615	6.053	1.127	0.260
Jagsi R. et al	3.600	2.328	5.567	5.759	0.000
Sharma	1.310	0.502	3.421	0.551	0.581
	1.318	0.848	2.049	1.229	0.219

Statistics for each study

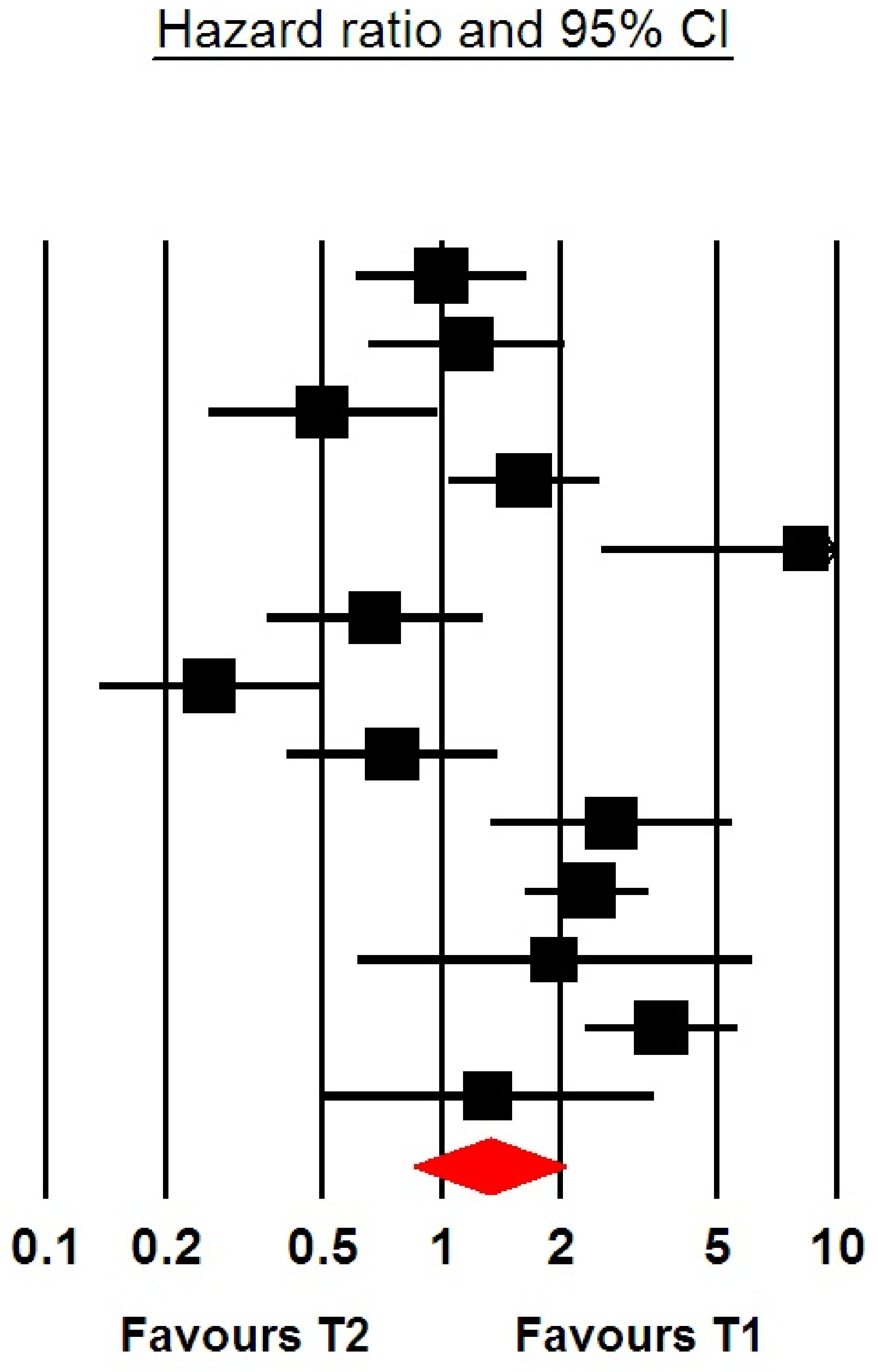

Study name	Statistics for each study				
	Hazard ratio	Lower limit	Upper limit	Z-Value	p-Value
Selz et al	0.360	0.119	1.085	-1.815	0.070
Abi-Raad et al	1.470	0.826	2.618	1.309	0.191
Yildrim E. et al	3.220	0.867	11.959	1.747	0.081
Truong P.T. et al	0.700	0.320	1.531	-0.894	0.372
Li J.L. et al	0.980	0.520	1.848	-0.062	0.950
Mamtani A. et al	1.060	0.446	2.518	0.132	0.895
	1.046	0.761	1.438	0.276	0.783

Hazard ratio and 95% CI

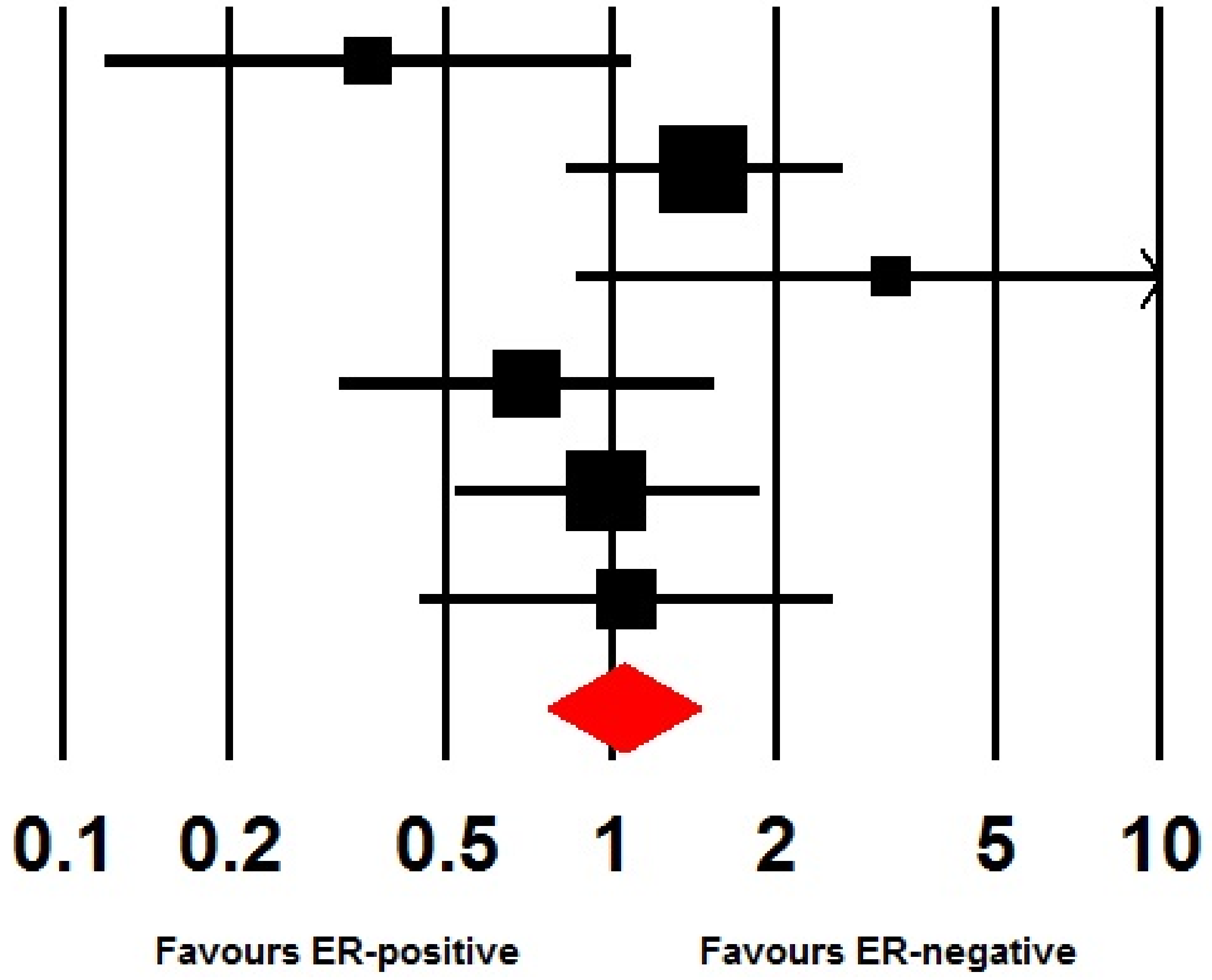

Study name	Hazard ratio	Lower limit	Upper limit	Z-Value	p-Value
Selz et al	0.360	0.119	1.085	-1.815	0.070
Abi-Raad et al	1.470	0.826	2.618	1.309	0.191
Yildrim E. et al	3.220	0.867	11.959	1.747	0.081
Truong P.T. et al	0.700	0.320	1.531	-0.894	0.372
Li J.L. et al	0.980	0.520	1.848	-0.062	0.950
Mamtani A. et al	1.060	0.446	2.518	0.132	0.895
	1.046	0.761	1.438	0.276	0.783

(Spanning header above the statistics columns: Statistics for each study)

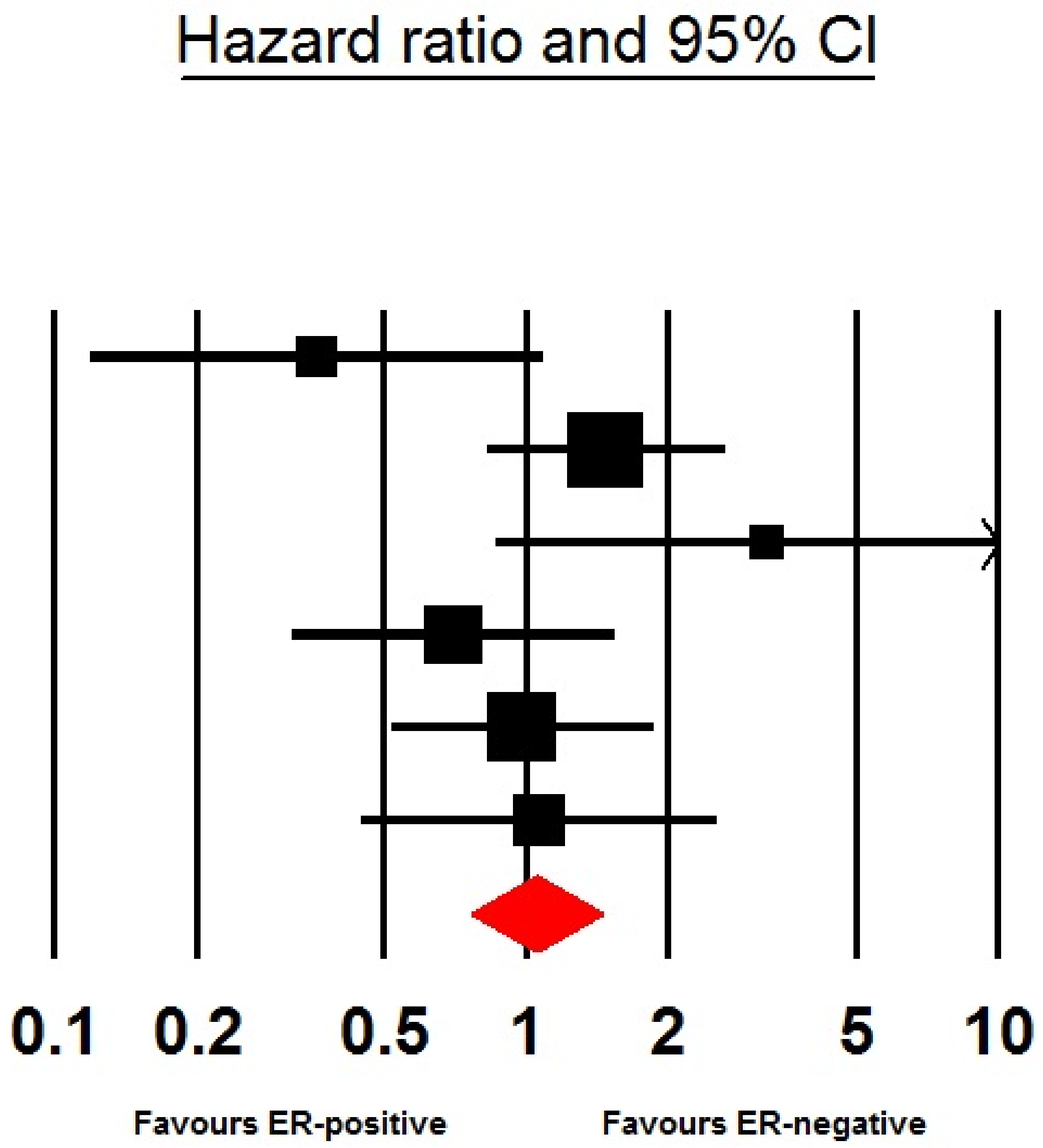

Supplement: Supplementary file 1 [file bsr20181853_Supp1.pdf]
